# Age and sex associate with outcome in older AML and high risk MDS patients treated with 10-day decitabine

**DOI:** 10.1038/s41408-023-00850-6

**Published:** 2023-06-19

**Authors:** Jacobien R. Hilberink, Isabelle A. van Zeventer, Dana A. Chitu, Thomas Pabst, Saskia K. Klein, Georg Stussi, Laimonas Griskevicius, Peter J. M. Valk, Jacqueline Cloos, Arjan A. van de Loosdrecht, Dimitri Breems, Danielle van Lammeren-Venema, Rinske Boersma, Mojca Jongen-Lavrencic, Martin Fehr, Mels Hoogendoorn, Markus G. Manz, Maaike Söhne, Rien van Marwijk Kooy, Dries Deeren, Marjolein W. M. van der Poel, Marie Cecile Legdeur, Lidwine Tick, Yves Chalandon, Emanuele Ammatuna, Sabine Blum, Bob Löwenberg, Gert J. Ossenkoppele, Dutch-Belgian Hemato-Oncology Cooperative Group (HOVON), D. A. Chitu, S. K. Klein, L. Griskevicius, P. J. M. Valk, J. Cloos, A. A. van de Loosdrecht, D. Breems, D. van Lammeren-Venema, R. Boersma, M. Jongen-Lavrencic, M. Söhne, R. van Marwijk Kooy, D. Deeren, M. W. M. van der Poel, M. C. Legdeur, L. Tick, E. Ammatuna, B. Löwenberg, G. J. Ossenkoppele, G. Huls, Swiss Group for Clinical Cancer Research (SAKK), T. Pabst, G. Stussi, M. Fehr, M. G. Manz, Y. Chalandon, S. Blum, Gerwin Huls

**Affiliations:** 1 Department of Hematology, University Medical Center Groningen, Groningen, the Netherlands; 2 Department of Hematology, HOVON Data Center, Erasmus MC Cancer Institute, Rotterdam, the Netherlands; 3 Department of Oncology, University Hospital, Inselspital, and University of Bern, Bern, Switzerland; 4 Department of Internal Medicine, Meander Hospital Amersfoort, Amersfoort, the Netherlands; 5 Department of Hematology, Oncology Institute of Southern Switzerland, Ospedale Regionale, Bellinzona, Switzerland; 6 Hematology, Oncology and Transfusion Medicine Center, Vilnius University Hospital Santaros Klinikos, Vilnius University, Vilnius, Lithuania; 7 Department of Hematology, Erasmus University Medical Center and Erasmus MC Cancer Institute, Rotterdam, the Netherlands; 8 Department of Hematology, Amsterdam UMC, VU University Medical Center, Cancer Center Amsterdam, Amsterdam, the Netherlands; 9 Department of Hematology, ZNA Stuivenberg/Middelheim, Antwerp, Belgium; 10 Department of Hematology, Hagaziekenhuis, Den Haag, the Netherlands; 11 Department of Hematology, Amphia Hospital, Breda, the Netherlands; 12 Department of Medical oncology and Hematology, Kantonsspital St. Gallen, St. Gallen, Switzerland; 13 Department of Hematology, Medical Center Leeuwarden, Leeuwarden, the Netherlands; 14 Department of Medical Oncology and Hematology, Universitätsspital Zurich, Zurich, Switzerland; 15 Department of Hematology, Antonius Hospital, Nieuwegein, the Netherlands; 16 Department of Hematology, Isala Hospital, Zwolle, the Netherlands; 17 Department of Hematology, AZ Delta Roeselare, Roeselare, Belgium; 18 Department of Internal Medicine, Division of Hematology, GROW School for Oncology and Developmental Biology, Maastricht University Medical Center, Maastricht, the Netherlands; 19 Department of Hematology, Medical Spectrum Twente, Enschede, the Netherlands; 20 Department of hematology, Maxima Medical Center, Veldhoven, the Netherlands; 21 Division of hematology, University Hospital Genève and Faculty of Medicine, University of Genève, Genève, Switzerland; 22 Service and Central Laboratory of Hematology, Department of Oncology and Department of Laboratory Medicine and Pathology, Lausanne University Hospital (CHUV), Lausanne, Switzerland

**Keywords:** Acute myeloid leukaemia, Myelodysplastic syndrome, Chemotherapy

## Abstract

Treatment choice according to the individual conditions remains challenging, particularly in older patients with acute myeloid leukemia (AML) and high risk myelodysplastic syndrome (MDS). The impact of performance status, comorbidities, and physical functioning on survival is not well defined for patients treated with hypomethylating agents. Here we describe the impact of performance status (14% ECOG performance status 2), comorbidity (40% HCT-comorbidity index ≥ 2), and physical functioning (41% short physical performance battery < 9 and 17% ADL index < 6) on overall survival (OS) in 115 older patients (age ≥ 66 years) treated on a clinical trial with a 10-day decitabine schedule. None of the patient-related variables showed a significant association with OS. Multivariable analysis revealed that age > 76 years was significantly associated with reduced OS (HR 1.58; *p* = 0.043) and female sex was associated with superior OS (HR 0.62; *p* = 0.06). We further compared the genetic profiles of these subgroups. This revealed comparable mutational profiles in patients younger and older than 76 years, but, interestingly, revealed significantly more prevalent mutated ASXL1, STAG2, and U2AF1 in male compared to female patients. In this cohort of older patients treated with decitabine age and sex, but not comorbidities, physical functioning or cytogenetic risk were associated with overall survival.

## Introduction

Acute myeloid leukemia (AML) is a heterogeneous disease with regard to tumor biology, clinical characteristics and outcomes with the currently available treatments [1]. The optimal treatment for older AML patients in daily clinical practice remains challenging and the choice of therapy is informed by disease characteristics (cytogenetic and molecular abnormalities), patients characteristics (age, performance, comorbidity, cognitive function) and preference of the patient [2, 3]. Many of the same challenges are encountered in the treatment of patients with high-risk myelodysplastic syndrome (MDS). Until very recently the anti-leukemic treatment options consisted of either intensive chemotherapy (anthracycline combined with cytarabine, known as ‘3 + 7’), hypomethylating agents (HMA)(azacitidine, decitabine) or low dose cytarabine. The addition of venetoclax to the HMAs azacitidine and decitabine has been shown to significantly improve median OS, from 9.6 to 14.7 months, and diverse combinations of classic chemotherapeutic agents with new targeted drugs may enter the therapeutic playing field in the near future [4].

Selection of the optimal treatment for older patients requires additional attention for treatment tolerance and life expectancy, estimated by evaluation of generally accepted prognostic factors like performance status, comorbidities, physical function and cognition [2, 5]. Several studies have shown the prognostic value of performance status prior to the start of intensive chemotherapy [6–9]. In addition, comorbidity burden (Charlson comorbidity index > 1 or hematopoietic cell transplantation comorbidity index (HCT-CI ≥ 3)) has been shown to be associated with lower remission rates, increased early mortality and decreased survival for patients treated with intensive chemotherapy [10–14]. Limited data are available on the impact of patient related factors on outcome when patients are treated with lower intensity therapies, usually containing a hypomethylating agent. A randomized study evaluating survival in patients treated with azacitidine or conventional care regimens (including intensive chemotherapy) found performance status to be associated with survival, but did not consider other patient-related factors [15]. In addition, patients with an impaired performance were found to have improved survival when treated with decitabine compared to supportive care [16]. However, randomized data in the setting of lower-intensity therapies are limited.

To more adequately assess vulnerabilities in older patients, beyond performance status and comorbidities, geriatric assessment is attracting attention. Geriatric assessment refers to a comprehensive approach assessing multiple patient characteristics (i.e. physical function, comorbid disease(s), cognitive function, psychological state, social support, polypharmacy, and nutritional status) to help characterize individual patient complexity and aims to discriminate between fit, vulnerable and frail patients. In older patients with AML treated with intensive chemotherapy the short physical performance battery (SPPB) and the modified minimal mental scale (3MS), both generally included in geriatric assessments, appeared to predict for survival [17, 18]. Physical performance, fatigue, and cognitive impairment were also associated with survival in a sub-analysis of 107 AML and MDS patients treated with non-intensive therapies (i.e., HMA or only best supportive care) [19]. Recently, it has been shown in a subset of patients enrolled in the CALGB 11002 trial, of whom 82 out of 165 patients performed baseline geriatric assessment, that physical functioning and cognition are associated with 6-month mortality and survival among older patients with AML treated with non-intensive chemotherapy (i.e., 10-day decitabine ± bortezomib) [20]. Further randomized data that explore comprehensive patient-assessment strategies to guide treatment (low intensity therapies vs intensive therapies) are not available.

In the prospective randomized HOVON135 trial (10-day decitabine vs 10-day decitabine + Ibrutinib) we evaluated in detail the impact of age, sex, performance status, comorbidity, SPPB, and activities of daily living (ADL) index on survival [21].

## Methods

### Study design & patient selection

This analysis was done in the context of the phase II multicenter HOVON135 clinical trial (EudraCT 2015-002855-85). The HOVON135 trial prospectively included 144 patients older than 65 years, diagnosed with AML or high-risk MDS without previous therapy, and not eligible for standard chemotherapy. Patients with secondary AML defined as AML after an antecedent hematological disease and therapy-related AML were eligible for inclusion. Patients were considered not eligible for intensive chemotherapy if they had a HCT-CI ≥ 3 or if the patients declined to receive intensive chemotherapy. Patients were randomized to receive 10-day decitabine + ibrutinib, ibrutinib given sequentially with decitabine starting the day after the last dose of decitabine until the day before the start of the next cycle, or 10-day decitabine alone [21]. Detailed information on trial design has previously been published [21]. This study was approved by the Medical Ethical committee of the University Medical Centre Groningen, and all participants provided informed consent in accordance with the Declaration of Helsinki.

### Objective physical function assessment

Assessment of physical functioning was performed at inclusion of the study. It consisted of the Short Physical Performance Battery (SPPB) and the Katz Index of Independence in Activities of Daily Living (ADL index). The SPPB is a validated tool to assess lower extremity function in older populations [22]. It comprises a short walk, repeated chair stands, and a balance test. Each item is scored ranging from 0 to 4 (0=unable to complete the exercise; 4 = highest performance level), with a total ranging from 0 to 12. A cut-off score of 9 was used to differentiate between patients with impaired and unimpaired physical performance [17, 18, 23]. The ADL index is a validated assessment tool for a person’s ability to perform daily self-care tasks, with a score of 6 reflecting independence and scores below 6 suggesting dependence [24]. In addition, the Eastern Cooperative Oncology Group (ECOG) performance status was recorded and comorbidity burden was scored according to the Hematopoietic Cell Transplantation Comorbidity Index (HCT-CI) [25, 26].

### Molecular analyses

With real-time PCR CBFB-MYHII, RUNX1-RUNX1T1 and FLT3 internal tandem duplications were detected on bone marrow aspiration samples or peripheral blood samples taken at diagnosis. Targeted next generation sequencing (NGS) was performed for 54 frequently mutated genes in hematologic malignancies with the Illumina TruSight Myeloid Sequencing panel, as previously described [21]. Cytogenetic risk groups were defined according to the European LeukemiaNet (ELN) 2017 AML risk classification [27].

### Statistical analysis

Descriptive statistics were used to characterize the study cohort. Overall survival (OS) was defined from date of study inclusion to date of death or last follow-up. Univariable Cox regression analysis was performed to calculate hazard ratios (HRs) of the SPPB score, the ADL index, HCT-CI score, and ECOG performance score at diagnosis. With multivariable Cox regression analysis the HRs were adjusted for age, sex, cytogenetic risk and AML or MDS diagnosis. Additionally, Kaplan-Meier curves were estimated for subgroups of the SPPB score, the ADL index, HCT-CI score, and ECOG performance score, and for cytogenetic risk, sex and age. Statistical comparison of mutation frequencies and clonal characteristics between sex and age subgroups was done using Fisher’s exact test and Mann-Whitney U tests. Odds ratios (OR) and 95% CI (confidence intervals) for the association between age or sex subgroups and mutation frequencies are reported.

## Results

### Study population and baseline clinical characteristics

**Fig. 1:**
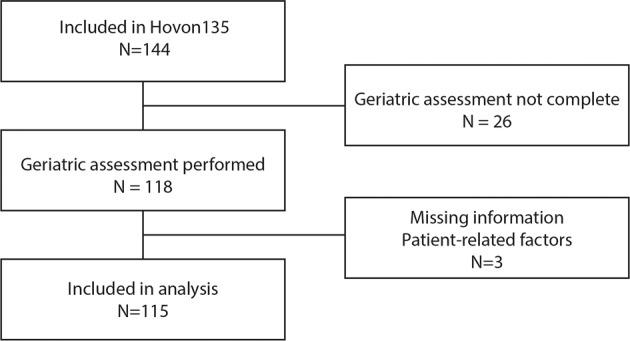
Flow diagram of patient cohort. One-hundred-forty-four patient were included in the Hovon135 study, for the current analysis 115 patient were included.

The complete cohort included 144 patients. Data regarding physical function assessment were available for 122 patients. Data on the ADL index was missing in 22 patients, and 21 patients did not have data on the SPPB (in 17 patients both the ADL index and the SPPB were missing). In addition, two patients had missing information on ELN risk classification and one patient had missing information on co-morbidities. One-hundred-fifteen fully evaluable patients were included in this analysis (Fig. 1). There was no difference in survival between the randomized treatment groups (i.e., decitabine with or without ibrutinib), therefore in the following analyses these groups were combined (Supplementary fig. 1) [21]. Median age at diagnosis was 76 years, 62% of patients were male, and the majority of patients was diagnosed with AML (Table 1). Sixty-two percent of patients had an adverse genetic risk profile according to ELN criteria. Physical function assessment scores are shown in Table 2. The majority of patients (83%) was independent in performing activities of daily living (i.e. ADL index score 6), but had an impaired performance status (ECOG score ≥ 1) (62%) or an increased comorbidity burden (HCT-CI ≥ 3) (40%). Forty-one percent of patients had a SPPB score below 9, indicating impairment in physical functioning.

**Table 1:** Characteristics of study population (*n* = 115).

*Characteristic*	No. (%)
Male	71 (62)
Age, median (IQR), y	76 (73–79)
Diagnosis	
- AML primary	70 (61)
- AML secondary	24 (21)
- MDS	21 (18)
ELN 2017 risk – *for AML patients*	
- Favorable	20 (17)
- Intermediate	16 (14)
- Adverse	58 (50)
IPSS-R risk score – *for MDS patients*	
≤ 6	16 (14)
> 6	5 (4)

**Table 2:** Baseline geriatric assessment scores (*n* = 115).

*Measure*	No. (%)
ECOG performance status	
- 0	44 (38)
- 1	55 (48)
- 2	16 (14)
HCT-CI	
- 0	33 (29)
- 1	24 (21)
- 2	12 (10)
- ≥ 3	46 (40)
SPPB	
- < 9	47 (41)
- ≥ 9	68 (59)
ADL index	
- < 6	20 (17)
- 6	95 (83)

### Association between patient-related factors and survival

**Fig. 2:**
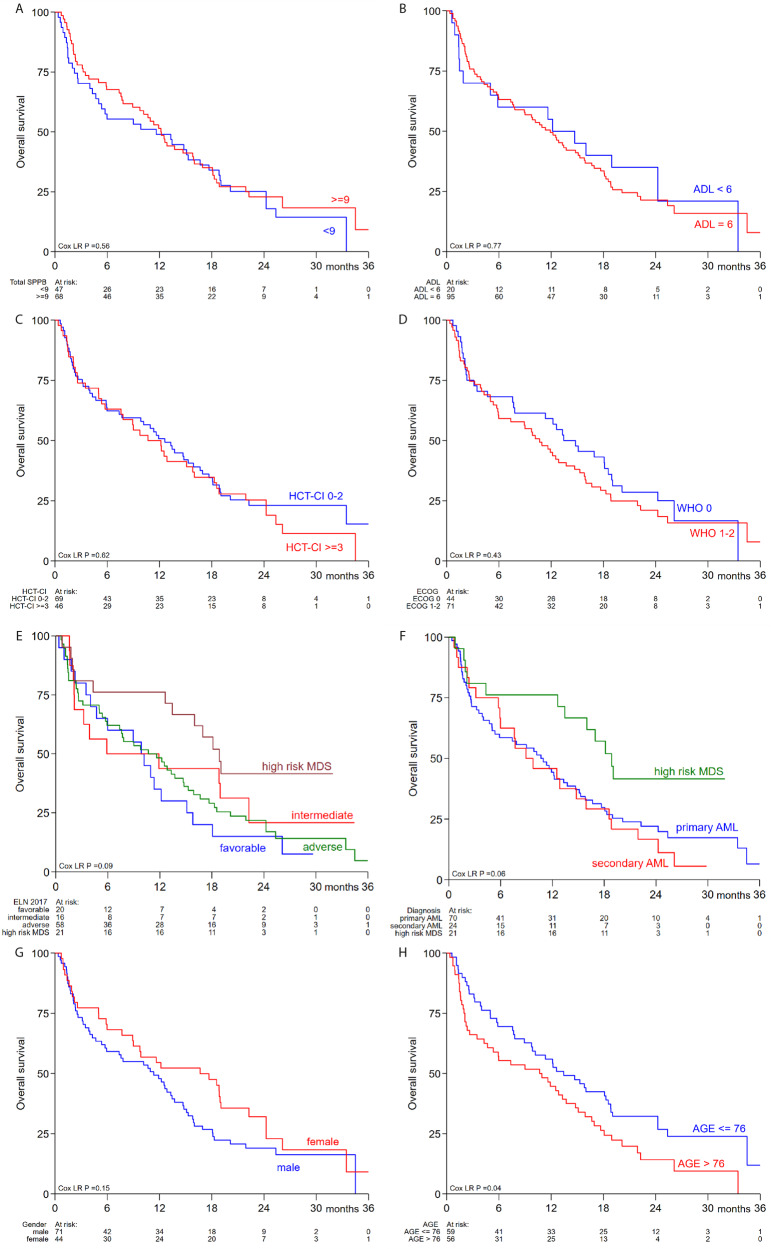
Kaplan-Meier estimates for overall survival stratified by patient-related variables. **A** OS stratified by SPPB score. **B** OS stratified by ADL score. **C** OS stratified by HCT-CI. **D** OS stratified by ECOG performance score. **E** OS stratified by ELN 2017 risk groups. **F** OS stratified by diagnosis. **G** OS stratified by sex. **H** OS stratified by age.

The median follow-up time was 11.3 months with a median overall survival of 11.5 months. The 30-day mortality was 5% and independent of patient-related factors. Notably, none of the patient-related variables (performance score, co-morbidities) nor variables included in physical function assessment at baseline (SPPB, ADL index) showed a significant association with survival in univariable or multivariable analysis (Table 3). Indeed, the Kaplan-Meier curves plotted for each variable were comparable between the subgroups (Fig. 2). Specifically, patients with a SPPB ≥ 9 had a median OS of 12.1 months and patients with a SPPB < 9 had a median OS of 11.6 months. When looking at ADL index, a median OS of 11.9 months was observed for patients with an ADL score of 6 versus 12.2 months for patients with an ADL score below 6. Patients with a HCT-CI < 3 had a median OS of 11.3 months (vs 12.2 months if ≥ 3) and patients with a ECOG PS 0 had a median OS of 13.4 months (vs 10.7 months if PS 1 or 2). Female patients had longer median OS compared to male patients although the difference was not significant (16.7 vs 11.3 months, respectively).

**Table 3:** Association between patient-related variables and survival.

	Univariable analysis		Multivariable analysis	
	Hazard ratio (95% CI)	*P*-value	Hazard ratio (95% CI)	*P*-value
Sex (female vs male)	0.73 (0.48–1.13)	0.16	0.62 (0.38–1.02)	0.061
ECOG performance status 1–2 (vs PS 0)	1.18 (0.77–1.81)	0.44	1.28 (0.80–2.05)	0.30
HCT-CI ≥ 3 (vs HCT-CI < 3)	1.11 (0.73–1.68)	0.61	0.91 (0.57–1.45)	0.63
SPPB ≥ 9 (vs SPPB < 9)	0.87 (0.57–1.31)	0.50	0.85 (0.54–1.32)	0.48
ADL index 6 (vs ADL < 6)	1.08 (0.63–1.86)	0.77	0.91 (0.49–1.68)	0.76
Age > 76 years (vs ≤ 76)	1.55 (1.02–2.34)	0.039	1.58 (1.01–2.46)	0.043
Diagnosis – *(relative to primary AML)*				
- Secondary AML	1.14 (0.69–1.86)	0.61	1.21 (0.71–2.07)	0.49
- MDS	0.53 (0.28–0.99)	0.048	0.45 (0.21–0.97)	0.040
ELN 2017 risk – (relative to favorable)				
- Intermediate	0.68 (0.33–1.43)	0.31	0.68 (0.32–1.46)	0.32
- Adverse	0.82 (0.48–1.41)	0.47	0.71 (0.40–1.25)	0.24
- MDS	0.42 (0.20–0.89)	0.023

When looking specifically at the SPPB scores at baseline, a group of patients (*n* = 12) could be identified with very low SPPB scores ( ≤ 4), indicating severely impaired physical functioning. This subgroup did not have significantly reduced OS (*p* = 0.79). In addition the Kaplan-Meier survival estimates of the subgroup with SPPB score 5–8 was comparable to the subgroup with SPPB score 9–12 (Supplementary fig. 2).

Additionally, the effect of cytogenetic risk, classified by ELN 2017 criteria, on survival was evaluated. Survival was not significantly different between AML patients with a favorable, intermediate or adverse cytogenetic risk. All three ELN risk groups had worse overall survival compared to high-risk MDS patients (Fig. 2E). Consistently, cytogenetic risk was not significantly associated with survival in univariable or multivariable analysis (Table 3). Further, the effect of primary versus secondary AML on survival was evaluated and no difference was observed (Fig. 1F; Table 3). High-risk MDS patients had superior survival compared to AML patients (HR 0.53, *p* = 0.048).

### Age and survival

Higher age (i.e., above median age of 76 years) was significantly associated with decreased survival in univariable analysis. This effect remained significant in multivariable analysis after adjusting for the patient-related factors (sex, performance score, co-morbidities, SPPB and ADL index) and genetic risk (HR 1.58, *p* = 0.043)(Table 3). The median OS was 13.4 months and 10.7 months for patients aged ≤ 76 or > 76 years respectively (Fig. 2H). The effect of age was not a surrogate for patient fitness or cytogenetic risk as these variables were included in the multivariable analysis and showed no effect on the risk estimate. An interaction between age and other patient-related factors was also considered but no significant interaction effect for age and SPPB or ADL index was observed.

### Age, sex and molecular subgroups

**Fig. 3:**
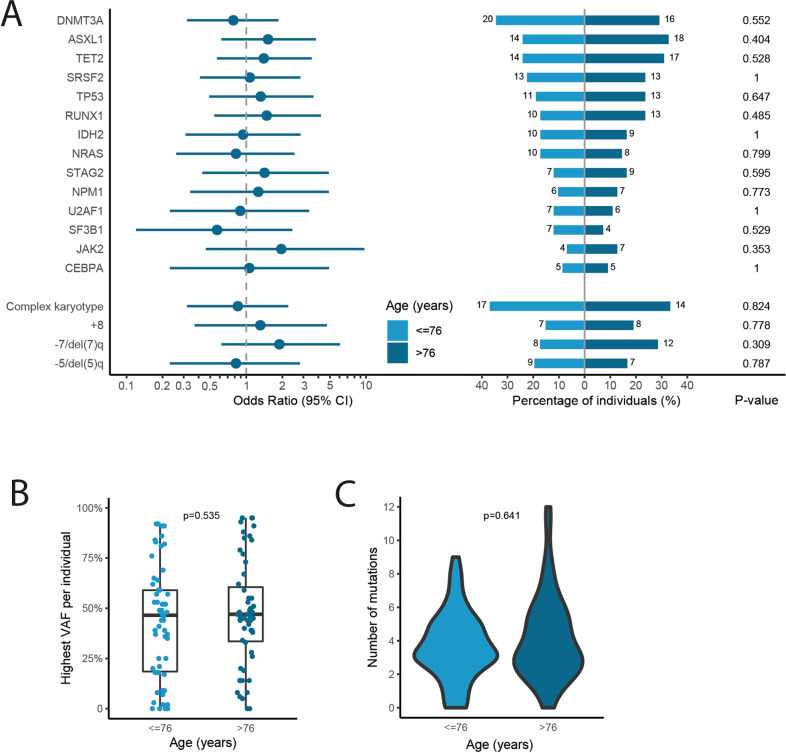
Mutational spectrum stratified by age group. **A** Forest plot indicating the OR and 95% CIs for the association between recurrently mutated genes and age > 76 years, and a pyramid plot displaying the proportion of patients and absolute numbers with a mutation in the recurrently mutated genes per age group. **B** Boxplot with the distribution of the highest VAF per individual stratified by age group. **C** Violin plot showing the distribution of the number of mutations per individual stratified by age group.

Since we were unable to identify patient related factors to explain the difference in survival amongst the age subgroups below and above the median age (i.e., ≤ 76 vs > 76 years), we compared the mutational profiles. Comprehensive molecular analysis was performed on patient material obtained from all included patients at diagnosis and extensive mutational data were available for 113 patients. Six patients (5%) had no detectable gene mutations. The highest variant allele frequency (VAF) per individual, the number of mutations per individual (median 3; range 0–12) and the number of mutated genes per individual (median 3; range 0–9) did not differ between patients aged ≤ 76 or > 76 years (Fig. 3B, C). With respect to the most common molecular abnormalities, evaluated by NGS, also no difference between patients aged ≤ 76 or > 76 years could be identified (Fig. 3A). Specifically there was no difference frequency of *TP53* mutations (11% vs 13%, *p* = 0.647) in patients aged ≤ 76 or > 76 years respectively.

**Fig. 4:**
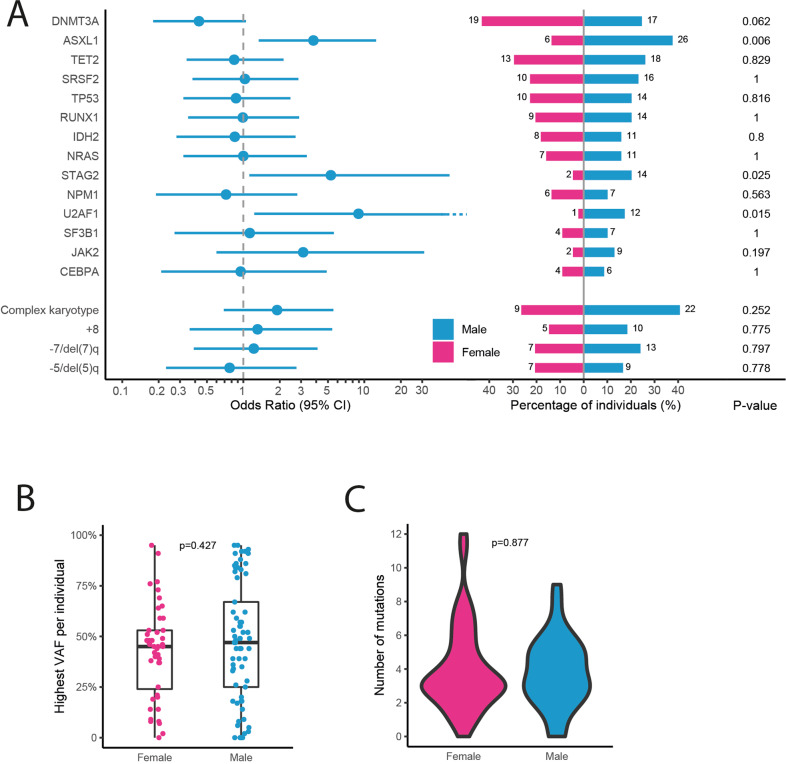
Mutational spectrum stratified by sex. **A** Forest plot indicating the OR and 95% CIs for the association between recurrently mutated genes and male sex, and a pyramid plot displaying the proportion of patients and absolute numbers with a mutation in the recurrently mutated genes for male and female patients. **B** Boxplot with the distribution of the highest VAF per individual in female and male patients. **C** Violin plot showing the distribution of the number of mutations per individual in female and male patients.

The trend that female sex was associated with superior survival was strengthened in the multivariable analysis (HR 0.62, *p* = 0.061)(Table 3). Although surprising at first instance the increased significance can be explained by the modifying effect of other factors influencing survival, namely MDS diagnosis (7 females, 14 males), on sex in univariable analysis. The almost significant impact of sex on survival prompted us to compare the mutational profiles in males and females in this study. Although the VAF per individual and the number of mutations per individual did not differ between male and female patients, interestingly the prevalence of mutations in certain genes (i.e., *ASXL1*, *STAG2*, and *U2AF1*) was significantly more frequent in male patients compared with female patients (Fig. 4A–C). Subsequently, we evaluated whether these gene mutations determined the observed inferior OS for male patients. In univariable analysis, though hampered by low numbers, mutations detected in either *ASXL1*, *STAG2*, or *U2AF1* were not significantly associated with survival (*ASXL1* HR 1.14 *p* = 0.57; *STAG2* HR 1.55 *p* = 0.12, *U2AF1* HR 0.94 *p* = 0.84). Also when these three mutations were combined, they were not significantly associated with survival (HR 1.15 *p* = 0.53). However, when the mutations were added in the multivariable analysis, the effect of sex on survival lost its borderline significance (HR 0.66, *p* = 0.118), suggesting that the difference in mutational profile between males and females, may at least partly explain the difference in survival between males and females.

## Discussion

The role of conventional prognostic factors for survival in older patients treated with low-intensity therapies is ambiguous. This study analysed the impact of performance status, comorbidity score, SPPB score and ADL score on survival in older patients with AML or high-risk MDS (who were considered unfit for intensive treatment or who refused intensive treatment) in the context of 10-day decitabine treatment. Of 115 patients included in this analysis, 41% had an SPPB score lower than 9, indicating physical impairment, and 40% had a comorbidity score of three or higher, confirming the ‘reduced fitness’ of our study population. However we found no significant association between any of these patient-related variables and survival. Surprisingly, in multivariable analyses only age at diagnosis (significant) and sex (borderline significant) were associated with survival.

Our results are in contrast with previously published results reporting a significant association between physical functioning and survival, which overruled chronologic age in predicting survival in two cohorts of older AML patients ( > 60 years) [17, 18]. However those studies were conducted in the context of treatment with intensive chemotherapy in contrast to our cohort which was treated with a hypomethylating agent. Further, many of the well-known prognostic factors have not been validated in older patient cohorts nor in patients treated with HMAs. The disparity in the impact of physical funtioning and chronologic age in both studies might be explained by the differences in anti-leukemic mechanism of action and in treatment-related toxicities. Indeed 30-day mortality in our cohort of older patients treated with decitabine was lower compared to cohorts of older patients treated with intensive chemotherapy [6, 17, 28, 29]. Increased co-morbidity burden and decreased performance score prior to intensive chemotherapy treatment have been associated with increased treatment-related toxicity and decreased survival [6–8, 10–12, 30, 31]. The association between objective physical functioning and survival found by Klepin et al. and Min et al. in patients treated with intensive chemotherapy is in line with this [17, 18]. Although evidence on the predictive value of geriatric assessment in AML patients treated with intensive chemotherapy is growing, the data in the context of less intensive therapies as yet has remained limited. In contrast to our findings, a recent study performed in a (biased) subset of a cohort of older patients treated with decitabine found a significant association between comorbidity score and survival and between cognition and survival. Decreased physical functioning was associated with increased 6-month mortality but not with overall survival [20]. Interestingly, patients diagnosed with high-risk MDS had better survival compared to patients diagnosed with AML. This finding seems contradictory with the recently revised diagnostic criteria for myeloid neoplasms emphasizing continuum between MDS and AML diagnoses and overruling the blast threshold [32].

In contrast to patients with AML treated with intensive chemotherapy, the association between the ‘traditional’ prognostic factors and HMA therapy is also less strong. Indeed in accordance with our results several studies have shown that patients classified as having ‘adverse’ risk AML based on ELN 2017 cytogenetic risk criteria don’t have significantly inferior survival after HMA treatment compared to ELN 2017 favorable and intermediate risk groups [15, 16, 33]. In line with this, the ELN 2017 classification did not provide prognostic infromation in the VIALE-A trial (comparing azacitidine with azacitidine + venetoclax) [34]. The median OS in our cohort, of which the majority had adverse cytogenetic characteristics, was comparable to the median OS reported for older patients after both intensive chemotherapy or HMA therapy [4, 15, 29, 35, 36]. Apparently the ELN 2017 risk classification, which was largely built using chemotherapy-treated patient datasets doesn’t maintain its prognostic impact in this patient cohort treated with a HMA. Our data suggest that all older patients, independent of patient-related factors (co-morbidity, physical functioning) or disease-related factors (ELN risk groups) can benefit of treatment with 10-day decitabine.

The age-effect in AML has been a widely discussed topic for many years. It has been hypothesized that age acts a surrogate marker for both patient-related factors and disease-related factors. Indeed it has been reported that the mutational profile differs between older and younger AML patients, with older patients harbouring more mutations associated with treatment-resistance and poor survival [5, 37–40]. Older patients also tend to have more comorbidities and impaired physical performance, which have been independently associated with treatment outcome [11, 12, 19, 31]. In line with this, some studies have shown the overruling effect of physical performance and cytogenetic risk on chronologic age when they were evaluated together in multivariable analyses [8, 17, 18]. However, in our analysis this was not the case, as age remained the variable with the strongest association with survival in our multivariable model, which also included sex, performance status, co-morbidities, SPPB score, ADL index and ELN risk. Apparently, in our cohort of patients treated with 10 days decitabine, the age-effect could not be explained by these patient derived variables. The availaibility of detailed molecular characterization of our cohort allowed us to investigate the possibility that the “age-effect” could be explained by a different mutational profile in the oldest patients. Therefore, we evaluated the mutational spectrum, number of mutations in patients, and number of mutated genes aged above or below 76 years and found no difference. Thus what comprises the effect of age, a mere number, in this cohort of AML patients treated with decitabine remains to be determined. We speculate that epigenetic mechanisms might play a role and may partly explain this age-effect.

Although it is widely known that the incidence of AML and MDS is higher in males compared to females, little is known about sex as a prognostic factor for survival. Our data show that older female patients tend to have a superior survival compared to older male patients after treatment with 10-day decitabine which is in accordance with previously published data [41]. In contrast to age, sex was associated with specific molecular abnormalities. Our data confirm recent manuscripts demonstrating that *ASXL1*, *U2AF1* and *STAG2* (encoded on the X-chromosome) are significantly more frequently mutated in male AML and MDS patients compared to female AML patients [38, 42–44]. Previously we have shown in the same cohort that patients with mutations in *STAG2*, *IDH2*, and *ASXL1* showed significantly reduced CR/CRi rates within 3 cycles of decitabine (12% for *STAG2*, 17% for *IDH2*, and 20% for *ASXL1*) [21]. Apparently, men more frequently have a mutational profile with lower chance of response to decitabine, which may explain the worse outcome of male patients in our study. Indeed, although hampered by low numbers, our data suggests that the difference in mutational profiles between males and females, at least partly, can explain the difference in survival between males and females. A recent paper published by the GenoMed consortium found some of the same sex-dependent mutations in MDS patients and showed that these mutations had a prognostic impact [44]. Furthermore, analyses of sex-specific differences in mutational spectrum of clonal hematopoiesis in a large population-based cohort showed enrichment for ASXL1, SF3BI, SRSF2 and U2AF1 in males [45].

The method of data collection, in the context of a prospective multicenter study, is a strength of this study since scores on objective physical performance tests did not influence the treatment decision. In addition, detailed molecular screening was available for the majority of included patients. Further, this trial confirms, what has been suggested by others, that older patients with AML and reduced fitness can be safely included in a clinical trial [46]. However, the number of patients in molecular subgroups was limited and objective physical performance assessment was not performed on all patients at baseline. It remains unclear whether patients declined to participate, weren’t able to due to impairment, or physical function assessment was simply forgotten.

In conclusion, not performance status, comorbidities or physical functioning but age and sex had impact on survival in patients treated with 10-day decitabine. Treatment with 10-day decitabine can benefit patients with ‘traditionally’ poor risk AML and MDS such as those with severe co-morbidities, low physical performance and adverse cytogenetic risk profile. Future research should continue to identify criteria that can enable the prediction of treatment response and treatment tolerance also for patients candidate for less intensive therapies as the therapeutic field moves towards personalized care.

## Supplementary information

Supplementary material

## Data Availability

The datasets generated during and/or analysed during the current study are not publicly available due to patient privacy concerns but are available from the corresponding author on reasonable request.
