# Supplementary material for: Age and sex associate with outcome in older AML and high risk MDS patients treated with 10-day decitabine
Source: Blood Cancer J. 2023 Jun 19;13(1):93. doi: 10.1038/s41408-023-00850-6 (PMC10279734; doi:10.1038/s41408-023-00850-6)
Supplement: Supplementary file 1 — Supplementary material [file 41408_2023_850_MOESM1_ESM.docx]

**Supplementary Data**

**
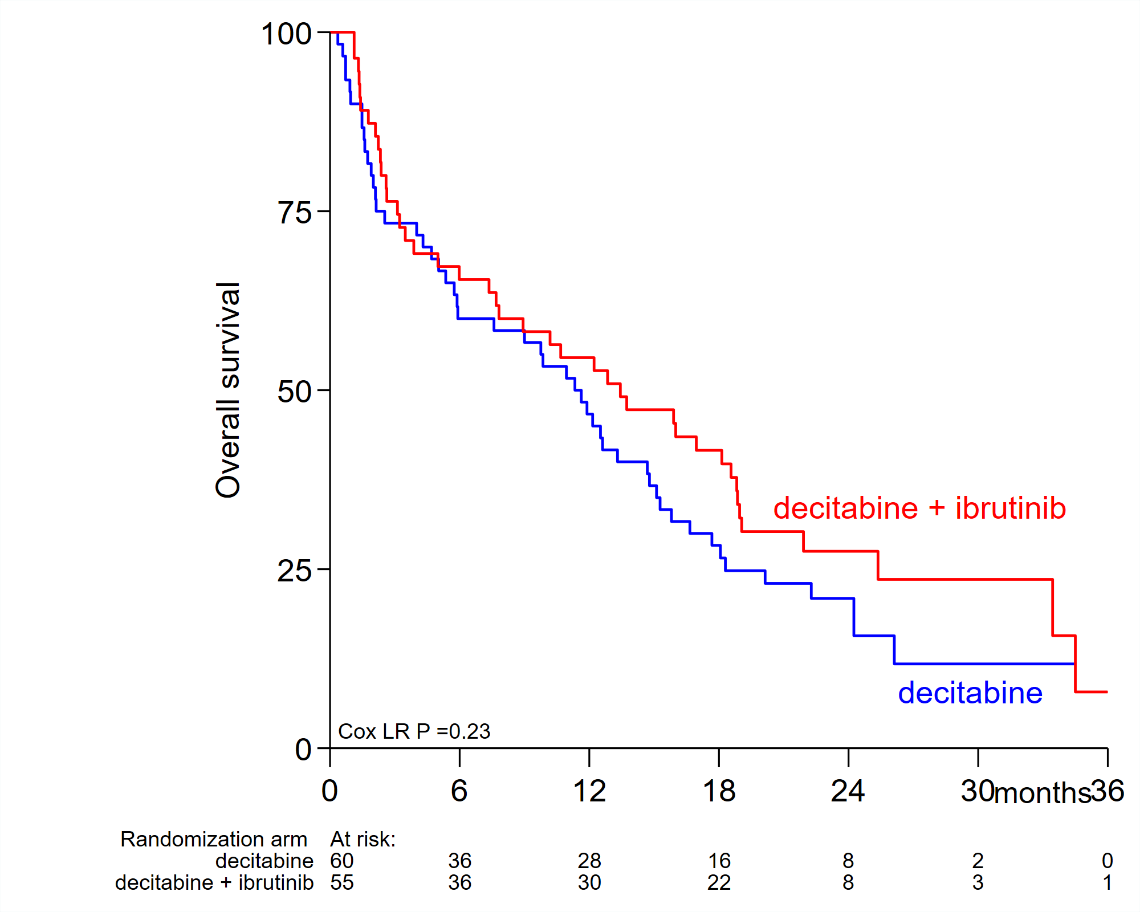
**

**Supplementary Figure 1.** **Kaplan-Meier estimates for OS in HOVON135 treatment arms.**

**
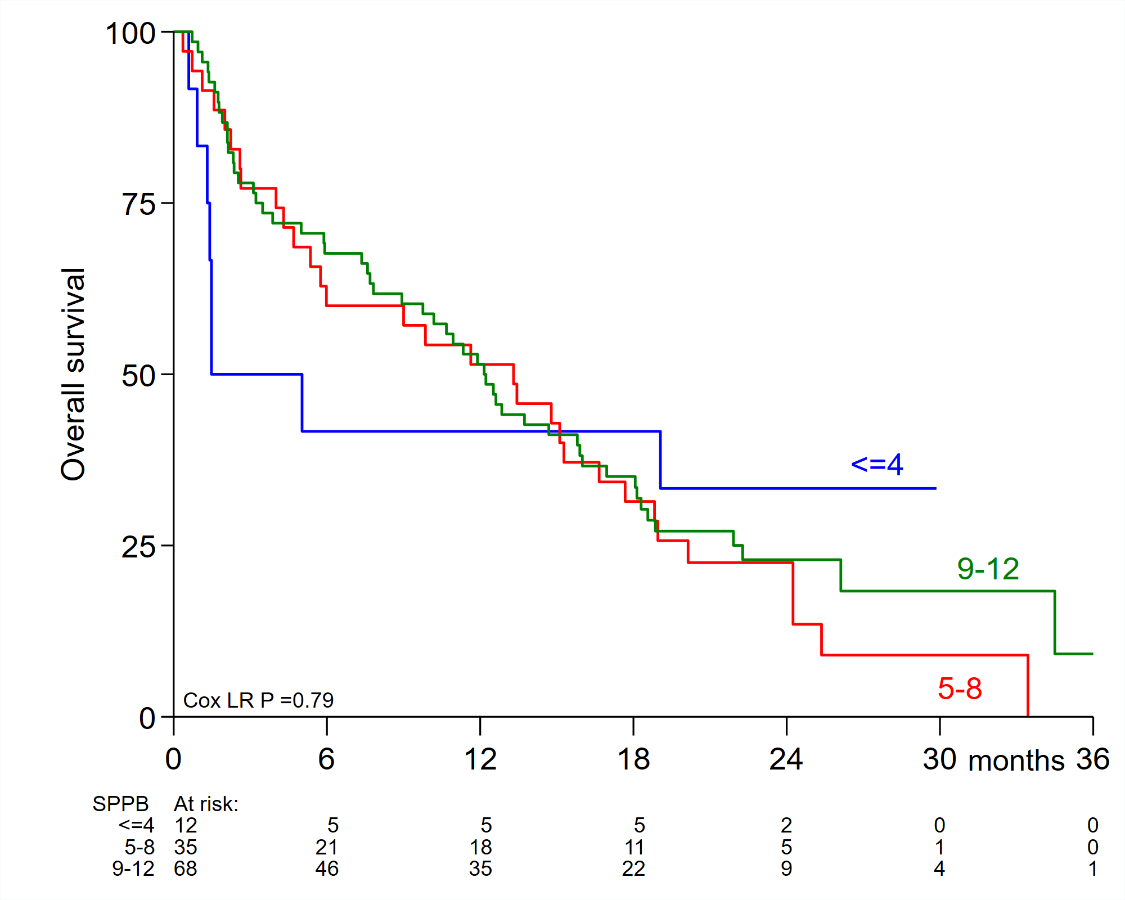
**

**Supplementary Figure 2. Kaplan-Meier estimates for OS stratified by SPPB score.** Scores 9-12 indicating no physical impairment, scores 5-8 indicating moderate physical impairment, and scores ≤ 4 indicating severe physical impairment.
